# Thrombin-Induced Calpain Activation Promotes Protease-Activated Receptor 1 Internalization

**DOI:** 10.1155/2017/1908310

**Published:** 2017-11-09

**Authors:** Alejandro Alvarez-Arce, Irene Lee-Rivera, Edith López, Arturo Hernández-Cruz, Ana María López-Colomé

**Affiliations:** Instituto de Fisiología Celular, Universidad Nacional Autónoma de México, México City, Mexico

## Abstract

The serine protease thrombin activates Protease-Activated Receptors (PARs), a family of G-protein-coupled receptors (GPCRs) activated by the proteolytic cleavage of their extracellular N-terminal domain. Four members of this family have been identified: PAR1–4. The activation of Protease-Activated Receptor 1(PAR1), the prototype of this receptor family, leads to an increase in intracellular Ca^+2^ concentration ([Ca^+2^]i) mediated by G_q11_*α* coupling and phospholipase C (PLC) activation. We have previously shown that the stimulation of PAR1 by thrombin promotes intracellular signaling leading to RPE cell transformation, proliferation, and migration which characterize fibroproliferative eye diseases leading to blindness. Within this context, the elucidation of the mechanisms involved in PAR1 inactivation is of utmost importance. Due to the irreversible nature of PAR1 activation, its inactivation must be efficiently regulated in order to terminate signaling. Using ARPE-19 human RPE cell line, we characterized thrombin-induced [Ca^+2^]i increase and demonstrated the calcium-dependent activation of *μ*-calpain mediated by PAR1. Calpains are a family of calcium-activated cysteine proteases involved in multiple cellular processes including the internalization of membrane proteins through clathrin-coated vesicles. We demonstrated that PAR1-induced calpain activation results in the degradation of *α*-spectrin by calpain, essential for receptor endocytosis, and the consequent decrease in PAR1 membrane expression. Collectively, the present results identify a novel *μ*-calpain-dependent mechanism for PAR1 inactivation following exposure to thrombin.

## 1. Introduction

The retinal pigment epithelium (RPE) is a monolayer of differentiated, quiescent cells located between the neural retina and the choroid. The RPE is the predominant component of the outer blood-retina barrier (BRB) and plays an essential role in the maintenance of the functional and structural integrity of the neural retina required for visual function. Among its functions, the RPE is involved in the transepithelial transport of nutrients, the storage and metabolism of vitamin A derivatives, the renewal of photoreceptor outer segments, and the ionic homeostasis of the subretinal space [1].

Under pathological conditions involving the alteration of the BRB due to ocular trauma, diabetic retinopathy, retinal detachment, retinal hemorrhage, or, importantly, retinal surgery, the RPE is exposed to serum-contained growth factors and proinflammatory agents including thrombin, generated by the activation of the coagulation cascade. Thrombin has been shown to promote the release of cytokines, chemokines, and growth factors by RPE cells [2]. This results in the proliferation, dedifferentiation, and migration of RPE and glia cells to the vitreous and the subsequent assembly of contractile membranes on both retinal surfaces, thus promoting retinal detachment and, eventually, the loss of vision [3]. The identification of molecular targets for the prevention of this outcome is still lacking.

Thrombin-induced intracellular signaling is triggered by Protease-Activated Receptors (PARs), a family of G-protein-coupled receptors (GPCRs) activated by the proteolytic cleavage of the extracellular N-terminal domain, which unmasks a fresh N-terminal sequence that functions as an intramolecular ligand. Four members of this family have been identified: PAR1, PAR3, and PAR4 activated by thrombin and PAR2 mainly activated by trypsin, tryptase, and other trypsin-like proteases, in addition to high concentrations of thrombin [4]. PAR1 is the prototype of this receptor family, and its cleavage by thrombin at the Arg41-Ser42 bond exposes a new N-terminus (^42^SFLLRN^47^) that acts as a tethered ligand which binds intramolecularly to the second extracellular loop of the receptor and triggers signaling. Synthetic ligands corresponding to the cleaved N-terminus can displace the tethered ligand from the binding site and fully activate PAR1 in an intermolecular mode. The coupling of PARs to GPCR G*α* subunits G_q11_*α*, G_12/13_*α*, and G_i_*α* has been linked to a wide array of physiologic responses. Particularly, PAR1 coupling to G_q11_*α* activates phospholipase C-*β* (PLC-*β*), which catalyzes the formation of inositol 1,4,5-trisphosphate (IP_3_) and diacylglycerol (DAG), leading to an increase in intracellular Ca^+2^ concentration ([Ca^+2^]i) [5].

Due to the irreversible nature of PAR1 activation by thrombin, PAR1 signaling and inactivation must be tightly regulated [6, 7]. Similar to classic GPCRs, PAR1 is rapidly desensitized by G-protein-coupled receptor kinase-mediated phosphorylation [8, 9] and *β*-arrestin binding, which uncouples the receptor from heterotrimeric G protein signaling [10]. Activated PAR1 is then internalized from the cell surface, sorted directly to lysosomes and degraded, which prevents continued signaling by previously activated receptors [11, 12]. These findings indicate that internalization and lysosomal sorting of PAR1 are important for regulating the magnitude and duration of G protein signaling. Studies by Paing et al. (2006) on this matter have shown that although *β*-arrestin binding is mainly responsible for PAR1 desensitization, it is not required for receptor internalization. Moreover, the mechanisms that control constitutive internalization of native, uncleaved receptors appear to differ from those controlling the internalization of activated receptors. Nonactivated PAR1 cycles constitutively between the plasma membrane and intracellular stores, thereby replenishing the cell surface following thrombin exposure, leading to rapid resensitization to thrombin signaling independent of de novo receptor synthesis [13].

Both the constitutive internalization and the internalization of activated PAR1 proceed through clathrin-coated vesicles [13–15]. Previous studies have shown that the clathrin adaptor protein complex 2 (AP-2) is essential for constitutive receptor internalization and cellular recovery of thrombin signaling [13]. The clathrin adaptor AP-2 is a heterotetrameric complex formed by *α*, *β*2, *μ*2, and *σ*2 adaptin subunits and has critical functions in the assembly and recruitment of cargo proteins to clathrin-coated pits. The *μ*2-adaptin subunit of AP-2 binds directly to tyrosine-based motifs within the cytoplasmic- (C-) tail domain of a number of GPCRs including PAR1 [16] and is required for constitutive internalization and cellular resensitization to thrombin [13]. AP-2 also regulates activated PAR1 internalization via recognition of distal C-tail phosphorylation sites rather than the canonical tyrosine-based motif and has been shown to depend on epsin-1 interaction with ubiquitinated PAR1 [13, 17]. The mechanism by which activated PAR1 is recruited to clathrin-coated pits is presently not clear, and whether internalization of PAR2, PAR3, and PAR4 proceeds through the same pathway as PAR1 or is regulated by distinct mechanisms remains to be established.

Calpains are a family of calcium-activated cysteine proteases that catalyze the limited proteolysis of a number of cellular proteins in eukaryotes [18, 19]. To date, more than a dozen calpain isoforms and multiple splice variants have been identified [20]. The best-characterized members of this family, *μ*-calpain (calpain 1) and m-calpain (calpain 2), are activated, respectively, by micro- and millimolar Ca^2+^ concentrations [21]. Both these calpains include a 78–80 kDa catalytic subunit encoded by the CAPN1 or CAPN2 genes [20] and share a common 28 kDa regulatory subunit (CAPNS1; formerly CAPN4) [22]. Calcium binding to EF-hand calcium binding domains present in both calpain subunits results in conformational changes which expose the catalytic domain in the large subunit, thus activating calpain [18]. *μ*-Calpain is present in an inactive form in the cytoplasm of nonstimulated cells. Upon stimulation, the increase in [Ca^+2^]i within the low micromolar range induces *μ*-calpain activation by autoproteolysis of the N-terminus, which exposes the catalytic site [23].

Calpains are involved in multiple cellular processes such as cell migration, actin cytoskeleton remodeling, and apoptosis [18]. Among these functions, calpains regulate the internalization of membrane proteins through clathrin-coated vesicles [24], such as epidermal growth factor receptor (EGFR) [25], transferrin receptor (RTF) [26], Cystic Fibrosis Transmembrane Conductance Regulator (CFTR) [27], and Low Density Lipoprotein (LDL) [28] among others. This process has been shown to require the proteolysis of distinct structural proteins, including the cytoskeletal protein *α*-spectrin by calcium-activated *μ*-calpain [28, 29].

Using human RPE-derived ARPE-19 cell line, we analyzed thrombin-induced [Ca^+2^]i increase and demonstrated that the calcium-dependent activation of *μ*-calpain and the subsequent degradation of *α*-spectrin by calpain significantly decrease PAR1 expression at the cell membrane. These findings indicate that PAR1 inactivation by internalization is controlled by specific mechanisms, distinct from those promoting RPE cell transformation, and further support a role for thrombin in this process.

## 2. Materials and Methods

### 2.1. Reagents

All reagents used were cell culture grade. Thrombin, PAR1 agonist (SFLLRNPNDKYEPF), and anti-*α*-spectrin antibody (MAB1266) were purchased from Calbiochem/EMD Millipore (Billerica, MA, USA). PAR 3 (SFNGGP-NH_2_) and PAR4 (GYPGKF-NH_2_) agonist peptides were from Bachem (Torrance, CA, USA). PPACK (D-phenylalanyl-prolyl-arginyl chloromethyl ketone) was from Enzo Life Sciences (New York, NY, USA). DMEM/F12 was from Thermo Fisher Scientific (Waltham, MA, USA). Fetal bovine serum (FBS), Fluo-4 AM, Thapsigargin, EGTA, and Lanthanum (III) Chloride were purchased from Invitrogen, Life Sciences (Carlsbad, CA, USA). Calpain-Glo Protease Assay was from Promega (Madison WI, USA), and Epoxomicin and PAR1-Alexa 488 antibody (FAB3855G) were from R&D Systems, Inc. (Minneapolis, MN USA). Anti-PAR1 antibody (SC13503) was purchased from Santa Cruz Biotechnology (CA, USA). CY3 anti-mouse secondary antibody (115-165-003) was from Jackson ImmunoResearch (PA, USA). BAPTA-AM, acetyl-calpastatin, and PD150606 were from Tocris Bioscience (Minneapolis, MN, USA). All other reagents were from Sigma Aldrich (St. Louis MO, USA).

### 2.2. Cell Culture

ARPE-19 cell line derived from human RPE was used throughout (ATCC® CRL-2302™). Cells were grown in DMEM/F12 (Sigma Aldrich, St. Louis, MO, USA) supplemented with 15 mM HEPES, 14.2 mM NaHCO_3_, 0.5 mM sodium pyruvate, 0.005% penicillin, streptomycin, 0.01% neomycin, and 10% fetal bovine serum (FBS) pH 7.4. Cells were subcultured as suggested by ATCC. For all assays, cultures were serum-deprived for 24 hours prior to the experiment. Unless otherwise specified, experiments were carried in DMEM/F12 medium without FBS.

### 2.3. Measurement of Calpain Activity

Calpain-Glo Protease Assay (Promega, Madison, WI, USA) kit was used to determine the activity of m- and *μ*-calpain [30]. Cells were incubated for 30 minutes with 100 nM epoxomicin in order to inhibit proteasome activity. Cultures were then washed with phosphate buffered saline (PBS) and incubated for one hour with Suc-LLVY substrate peptide diluted with Calpain-Glo Buffer at 37°C. Cells were rinsed with PBS to remove unincorporated peptide and subsequently stimulated with 10 nM thrombin or 2.5 *μ*M PAR1-Agonist Peptide (AP) in DMEM/F12 medium at room temperature for 1 minute. The agonists were removed with PBS and cells were permeabilized using PBS 0.9% Triton X-100. Luciferin detection reagent (Promega, Madison, WI, USA) was then added for 5 min and luminescence was measured in a plate reader (Synergy HT, BioTek Instruments Inc., Winooski, VT, USA). Measurements for each experimental condition were registered at 20 s intervals for 2 minutes. Results are expressed as percent Relative Luminescence Units (% RLU). Data are the mean ± SEM of three independent experiments. Values for nonstimulated cultures maintained in serum-free DMEM/F12 (negative control) were set as 100%.

### 2.4. Anti-*α*-Spectrin Western Blot

Degradation of *α*-spectrin by calpain is one of the first events required for receptor internalization. Cells were stimulated for 1 minute with thrombin or PAR1-AP in serum-free DMEM/F12 at room temperature. Cultures were then rinsed with PBS and incubated for 15 minutes at 37°C in serum-free DMEM/F12. Cells were lysed in 50 mM Tris-HCl pH = 7.4, 150 mM NaCl, 10 mM EDTA, 0.1% SDS, 1% Triton X-100, 1% CHAPS, 0.5% NP40, 0.1% BSA, 10% protease inhibitor cocktail (Sigma Aldrich P8340), 40 mM *β*-glycerophosphate, and 10 mM sodium pyrophosphate. Protein concentration was determined using bicinchoninic acid assay (Sigma Aldrich) and 20 *μ*g of the total protein was used for protein immunodetection analysis. The lysates were solubilized in Laemmli buffer (0.75 mM Tris-HCl; pH 8.8, 5% SDS, 20% glycerol, 0.01% bromophenol blue, 10%  *β* mercaptoethanol), boiled for 5 min, resolved by SDS/PAGE (6.5%), and transferred onto nitrocellulose membranes (Amersham Biosciences). After blocking for 1 h at room temperature with 7.5% nonfat milk in Tween TBS, the membranes were probed with primary antibodies against *α*-spectrin (1 : 4000 in blocking buffer) overnight at 4°C. Secondary HRP-conjugated antibody (1 : 5000) was incubated for 1 h and membranes were developed using the Luminata Forte Western Chemiluminescent Substrate (Millipore, Billerica, MA, USA). Kodak® film images were digitized using an Alpha Digi-Doc system (Alpha Innotech, San Leandro, CA, USA), and densitometry analysis was performed using the ImageJ Software and normalized to control values. GAPDH immunodetection was used as loading control.

### 2.5. Intracellular Ca^+2^ Concentration ([Ca^2+^]i) Measurement

[Ca^+2^]i was determined as previously described [31]. Briefly, coverslips containing ARPE-19 cells were incubated with 2 *μ*M of the cell-permeable fluorescent Ca^2+^ indicator fluo-4 AM (Molecular Probes, Eugene, OR, USA) for 35 min at room temperature in Krebs-Ringer-Bicarbonate buffer (KRB; 118 mM NaCl; 2 mM KH_2_PO_4_; 4.7 mM KCl; 2.5 mM CaCl_2_; 1.4 mM MgSO_4_; 25 mM NaHCO_3_; 5.6 mM Glucose; pH 7.4). The coverslips were placed in a recording chamber (Mod. RC-25; Warner Instruments, Hamden, CT, USA) attached to the stage of an upright microscope (Nikon Eclipse 80i; Nikon Corp., Tokyo, Japan) and continuously superfused (3 ml/min) with KRB applied to the recording chamber by gravity-fed superfusion. Fluo-4 was excited at 488 nm with monochromatic light from an argon laser (Laser Physics, Reliant 100 s488, West Jordan, UT), coupled to a Yokogawa spin-disk confocal scan head (CSU10B, Yokogawa Electronic Co., Tokyo, Japan and Solamere Technology Group, Salt Lake city, USA). Emission light was captured with a 510 nm filter. Fluorescence images were acquired with a water-immersion, Nikon objective (20x, 0.5 NA), and a cooled digital CCD camera (Andor Technology iXon 897, Oxford Instruments, High Wycombe, UK) controlled by the iQ software (Andor iQ version 1.10.2). Fluorescence images were acquired at 10 ms exposure and 500 ms intervals. All intracellular Ca^2+^ imaging experiments were performed at room temperature (22–24°C). Image sequences were analyzed using Image J software (National Institutes of Health). The values obtained are expressed as Arbitrary Fluorescence Units (AFU). The area under each curve (AUC) was calculated using GraphPad PRISM 6.0 software (La Jolla, CA, USA). For those experiments performed in the absence of Ca^+2^, cells were superfused with Ca^+2^-free KRB; 0.25 mM EGTA or 100 *μ*M LaCl_3_ were included in some experiments as stated in the figure legends. Cells were stimulated with thrombin or PAR1, PAR3, or PAR4 APs for 1 minute.

### 2.6. Epifluorescence Microscopy

ARPE-19 cells were seeded onto 22 mm plates. Cells were serum-deprived for 24 hours and subsequently incubated for 30 min in the presence of BAPTA-AM (10 *μ*M) or the calpain inhibitors: calpastatin (1 *μ*M) or PD1506060 (100 *μ*M), followed by stimulation with either thrombin or PAR1-AP in DMEM/F12 medium for 5 minutes at room temperature. Subsequently, cells were rinsed with PBS and incubated for 15 min at 37°C. Blocking was performed with 5% BSA for 30 minutes and incubated for 4 hrs with Santa Cruz Anti-PAR1 antibody (1 : 500). Following primary antibody incubation, cells were fixed for 10 min with 4% paraphormaldehyde at 4°C. CY3 anti-mouse antibody was incubated for 1 hr (1 : 1000). Cells were washed 2x with PBS for 5 minutes. Nuclei were stained with Hoechst and further washed as before. Images were acquired with ACT-1 software in a Nikon microscope (Eclipse TE 2000-U) with DXM1200F camera and 40x objective (0.6 NA). Corrected Total Cell Fluorescence (CTCF) was calculated with ImageJ Software and normalized as percentage of control values.

### 2.7. Flow Cytometry

ARPE-19 cells were seeded onto 100 mm plates and serum-deprived for 24 hours. The cells were then detached using versene (5 mM Tris-HCl, 0.13 M NaCl, 0.5 M KCl, and 1.3 mM EDTA), and 5 × 10^5^ cells were used for each experimental condition. Suspended cells were incubated for 30 minutes with 10 *μ*M BAPTA-AM or calpain inhibitors (1 *μ*M calpastatin or 100 *μ*M PD1506060); the inhibitors were present throughout the experiment. Cells were stimulated for 5 minutes at room temperature, either with thrombin or with PAR1-AP in DMEM/F12 medium, rinsed with PBS, and blocked with 5% BSA-PBS. Cells were incubated with Anti-PAR1-Alexa 488 antibody for 3 hours at 4°C, and fluorescence was measured with Attune Acoustic Focusing flow cytometer (Thermo Fisher Scientific, USA). Data were analyzed using FlowJo, LLC 10.2 software (FlowJo, USA).

### 2.8. Statistical Analysis

Raw data for analyses were obtained from at least three independent experiments, as specified in the figure legends. Multiple comparison ANOVA and Tukey's post hoc test was applied to all results for statistical analysis. Prism V6.0 from GraphPad (La Jolla, CA, USA) was used.

## 3. Results

### 3.1. Thrombin Induces [Ca^+2^]i Increase

The effect of thrombin stimulation on RPE cell* [Ca*^*+2*^*]i* was determined using fluorescence microscopy, as described in the Methods. Although thrombin-induced increase in* [Ca*^*+2*^*]i* in RPE cells has been reported [32, 33], the characteristics of this response as a function of the intensity and duration of the stimulus, determinant for functional outcome, have not been analyzed.

**Figure 1:**
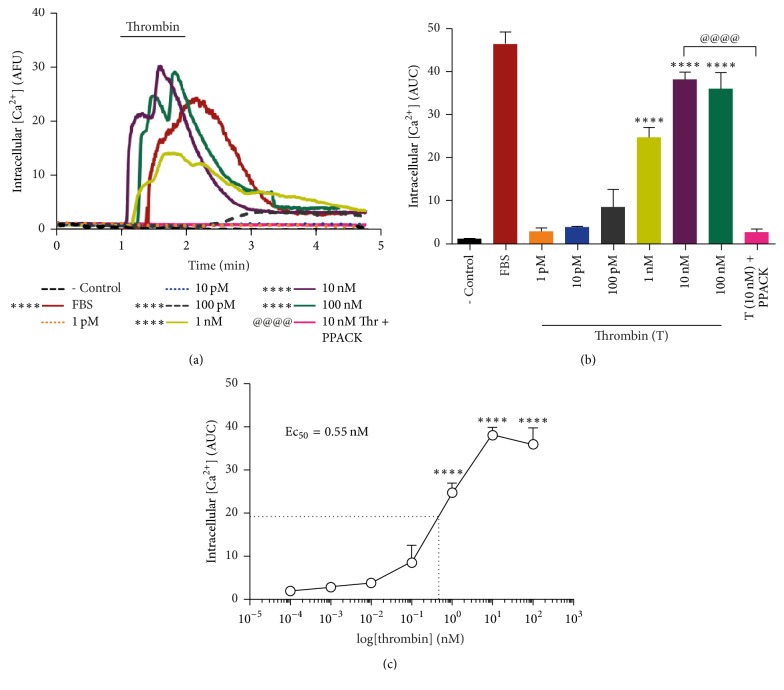
Thrombin induces a specific, dose-dependent increase in* [Ca*^2+^*]i*. Cells were serum-deprived for 24 hours prior to stimulation with increasing concentrations of thrombin.* [Ca*^*2+*^*]i* was monitored by fluorescence microscopy as described in the Methods. (a) Thrombin induces a transient, saturable* [Ca*^*2+*^*]i* increase, sustained for ~3 min.* [Ca*^*+2*^*]i* was plotted as a function of fluorescence intensity (Arbitrary Fluorescence Units (AFU)) over time (minutes). Specificity of thrombin effect was assessed by inclusion of the thrombin inhibitor PPACK (25 *μ*M). 10% FBS was included as positive control. (b) The area under the curves (AUC) plotted in (a) was calculated from the stimulation point up to the 4th minute of stimulation. (c) Thrombin Ec_50_ = 0.55 nM was calculated from the logarithmic transformation of data in (b). Results are expressed as the mean ± SEM of three independent experiments compared to nonstimulated cells (- Control). Multiple comparison ANOVA and Tukey's test: *α* = 0.001 (*∗∗∗∗*) referred to negative control or *α* = 0.001 (@@@@) referred to thrombin stimulation.

Results in Figure 1(a) show that stimulation with thrombin induces a transient, dose-dependent* [Ca*^*2+*^*]i* increase in RPE cells, sustained for ~3 min.* [Ca*^*2+*^*]i* increase was found to be dose-dependent from 100 pM thrombin concentration with maximum stimulation attained at 10 nM thrombin treatment, equivalent to calcium elevation induced by FBS (positive control). The specificity of the effect was demonstrated by inhibition upon the addition of the thrombin catalytic inhibitor PPACK (25 *μ*M) (Figures 1(a) and 1(b)). Calculation of the area under the curves (AUC) in (Figure 1(a)) from the stimulation time-point to the 4th minute is plotted as a function of fluorescence intensity (Arbitrary Fluorescence Units (AFU)) over time (Figure 1(b)). The Ec_50_ for thrombin effect was calculated from the logarithmic curve in (Figure 1(b)) and found to be Ec50 = 0.55 nM (Figure 1(c)).

### 3.2. Thrombin-Induced Increase in [Ca^2+^]i Is Mediated by PAR1

**Figure 2:**
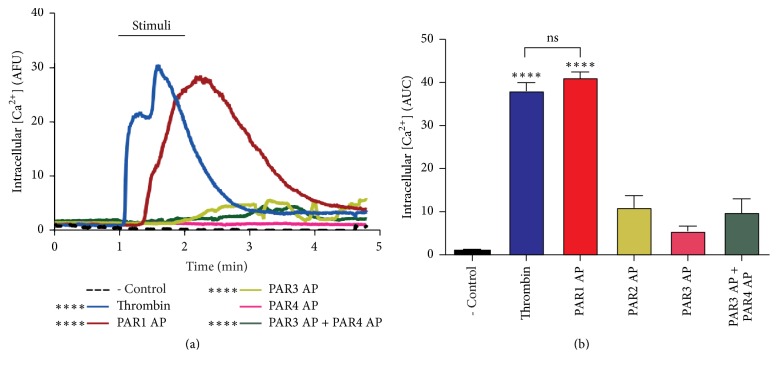
Thrombin-induced* [Ca*^+2^*]i *increase is mimicked by PAR1-AP. Cells were serum-deprived for 24 hours prior to stimulation with thrombin or PAR-APs.* [Ca*^*2+*^*]i* was assessed by fluorescence microscopy as described in Methods. (a) PAR1-AP (2.5 *μ*M) induced an increase in* [Ca*^*2+*^*]i*, comparable to thrombin.* [Ca*^*2+*^*]i* was plotted as a function of fluorescence intensity (Arbitrary Fluorescence Units (AFU)) over time (minutes). (b) The graph represents the area under the curves in (a). Thrombin (10 nM) stimulation was used as positive control. Results are expressed as the mean ± SEM of three independent experiments, compared to nonstimulated cells (- Control). Multiple comparison ANOVA and Tukey's test: *α* = 0.001 (*∗∗∗∗*) referred to negative control.

In order to identify the specific receptor mediating thrombin effect, we tested the effect of PAR-APs on* [Ca*^*2+*^*]i* using fluorescence microscopy as described in Methods.* [Ca*^*2+*^*]i* was plotted as a function of fluorescence intensity (AFU) over time (minutes). Results in Figure 2(a) show that only PAR1-AP (2.5 *μ*M) induced an increase in* [Ca*^*2+*^*]i* comparable to that elicited by 10 nM thrombin.  Figure 2(b) shows the calculated area under the curves (AUC) shown in Figure 2(a). These results demonstrate that PAR1 is responsible for* [Ca*^*+2*^*]i* increase induced by thrombin.

### 3.3. Calcium Release from Intracellular Pools Is the Main Source of Thrombin-Induced [Ca^2+^]i Increase

**Figure 3:**
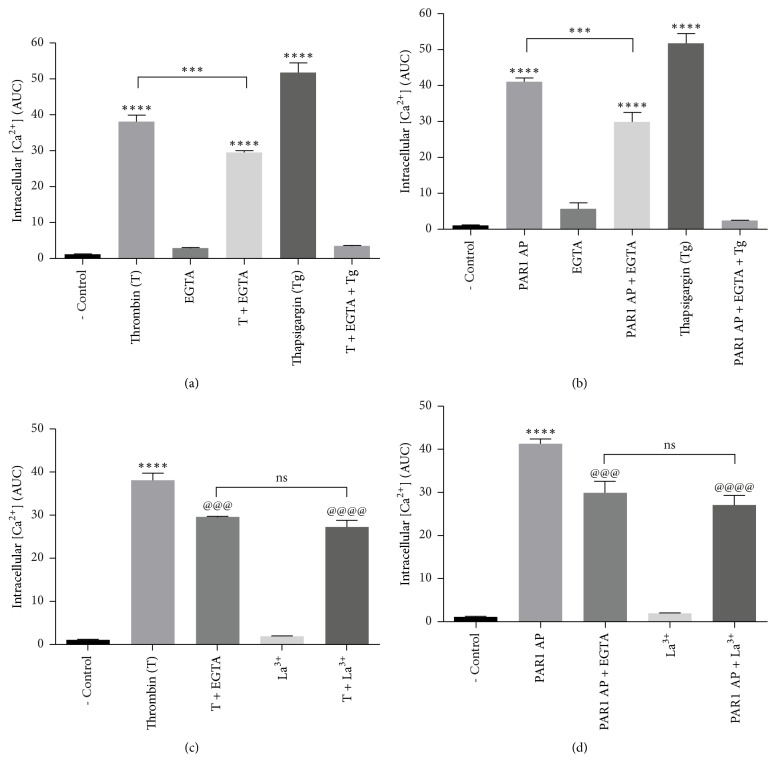
Thrombin promotes* [Ca*^2+^*]i *increase mainly by the release from intracellular pools. Serum-deprived cells were stimulated with thrombin or PAR1-AP.* [Ca*^*2+*^*]i* was determined by fluorescence microscopy as described in Methods. (a) Thrombin-induced* [Ca*^*+2*^*]i* increase is partially prevented by EGTA (0.25 mM) and abolished by thapsigargin (Tg; 2 *μ*M) + EGTA. (b) Thrombin-induced calcium release is mimicked by PAR1-AP. (c) Blockage of plasma membrane calcium channels by Lanthanum (La^3+^; 100 *μ*M) decreases thrombin-induced and (d) PAR1-AP-induced calcium increase to a similar extent as EGTA. Results are expressed as the area under the curve (AUC) compared to nonstimulated (NS) cells (negative control). Data are the mean ± SEM of three independent experiments. Multiple comparison ANOVA and Tukey's test: *α* = 0.001 (*∗∗∗∗*) or *α* = 0.01 (*∗∗∗*) referred to negative control. And *α* = 0.001 (@@@@) or *α* = 0.01 (@@@) referred to thrombin stimulation (10 nM) or PAR1 peptide agonist (2.5 *μ*M).

In order to determine the contribution of external and internal calcium pools to thrombin- and PAR1-induced* [Ca*^*+2*^*]i* increase, thrombin stimulation was carried in Ca^+2^ free medium containing 0.25 mM EGTA. As shown in Figure 3, thrombin and PAR1-AP responses were decreased by ~20% in this condition, indicating a minor contribution of extracellular calcium to thrombin-induced* [Ca*^*2+*^*]i* rise. In contrast, thrombin- and PAR1 AP-induced* [Ca*^*+2*^*]i* increase was inhibited by ~80% upon the inclusion of 2 *μ*M thapsigargin, known to deplete ER calcium stores (Figures 3(a) and 3(b)). These results were confirmed by the complete inhibition of thrombin effect by the joint inclusion of EGTA and thapsigargin. On this line, the store-operated calcium entry (SOCE) channels Orai and TRPC are activated by the depletion of intracellular Ca^2+^ stores. Since thrombin induces Gq/PLC*β* signaling, which is a physiological stimulus for store depletion, we tested the effect of the membrane Ca^2+^ channel inhibitor LaCl_3_, on PAR1-mediated* [Ca*^*+2*^*]i* increase.  Figure 3(c) shows that LaCl_3_ had a similar effect to that of EGTA, suggesting the possible participation of SOCE in PAR1-induced* [Ca*^*2+*^*]i* increase. Collectively, these data indicate that Ca^2+^ release from intracellular Ca^2+^ stores is the main source of* [Ca*^*2+*^*]i* increase induced by thrombin.

### 3.4. Thrombin Promotes Calpain Activity through PAR1 Activation

**Figure 4:**
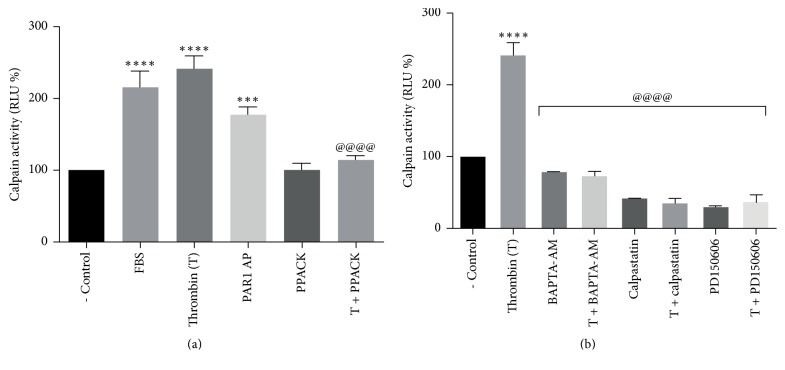
Thrombin activation of PAR1 stimulates the calcium-dependent activation of calpain. ARPE-19 cells were serum-deprived for 24 hours prior to stimulation for 1 min with 10 nM thrombin or 2.5 *μ*M PAR1-AP. PPACK, BAPTA-AM, and calpain inhibitors were included 30 minutes prior to stimulation. Calpain activation was measured as a function of luciferase activity as described in Methods. (a) Thrombin stimulates calpain activity by ~250% in a specific manner, prevented by PPACK. 10% FBS was included as positive control. PAR1-AP stimulates calpain activity by ~170% compared to negative control (- Control). (b) Calpain activation was prevented by the inclusion of BAPTA-AM (10 *μ*M), calpastatin (1 *μ*M), and PD150606 (100 *μ*M). Results in Relative Luminescence Units (RLU) are the mean ± SEM of three independent experiments. Values for nonstimulated cultures maintained in serum-free DMEM/F12 (- Control) were set as 100%. Multiple comparison ANOVA and Tukey's test: *α* = 0.001 (*∗∗∗∗*) or *α* = 0.01 (*∗∗∗*) referred to negative control; *α* = 0.001 (@@@@) referred to thrombin stimulation.

The effect of thrombin stimulation on calpain activity was assessed by the degradation of the synthetic calpain substrate peptide Suc-LLVY using Calpain-Glo Protease Assay, designed for measuring the activation of calpain isoforms *μ* and m [34]. Our results show that thrombin stimulation increases calpain activity by ~250% over control level. Activation appeared to be thrombin-specific, since it was prevented by the catalytic thrombin inhibitor PPACK (Figure 4(a)). In order to identify PAR1 as the mediator of thrombin effect, we tested the effect of PAR1-AP on calpain activity. Results showed that PAR1-AP stimulated calpain activity by ~170% (Figure 4(a)).

To establish if calpain activation by thrombin depends on thrombin-induced* [Ca*^*+2*^*]i* increase, cells were loaded with the selective cell-permeant Ca^2+^ chelator BAPTA-AM prior to thrombin stimulation. Results in Figure 4(b) show that inclusion of BAPTA-AM (10 *μ*M) completely prevented thrombin-induced calpain activation. Calpain activity was abolished by the calpain endogenous inhibitor calpastatin (1 *μ*M) and by the synthetic inhibitor PD150606 (100 *μ*M), thus confirming the specificity of the effect (Figure 4(b)). These data demonstrate that thrombin-induced [Ca^+2^]i increase promotes calpain activation through the activation of PAR1.

### 3.5. Activation of Calpain by Thrombin Stimulation Promotes *α*-Spectrin Proteolysis

Spectrin, an extended rod-shaped tetramer composed of two *α* and two *β* subunits, forms a cytoskeletal meshwork with actin and other accessory proteins lining the cytoplasmic surface of most cell membranes, thus providing a mechanism for restricting the free diffusion of transmembrane proteins [35]. Limited proteolysis of *α*-spectrin by *μ*-calpain [36] prevents spectrin interaction with actin [37] and also spectrin association with membrane-binding sites [38] allowing protein endocytosis [28]. Based on this evidence, we tested the effect of thrombin stimulation on *α*-spectrin proteolysis.

**Figure 5:**
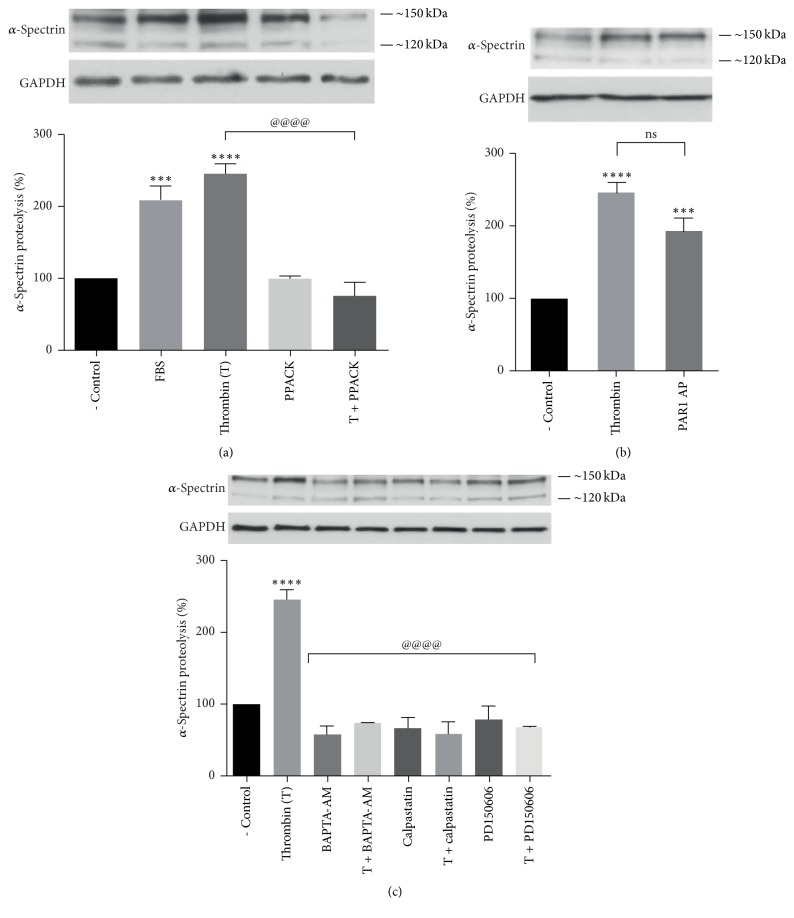
Thrombin induces*α-spectrin *degradation through PAR1-mediated *Ca*^2+^*-*dependent activation of calpain. Serum-deprived ARPE-19 cells were stimulated for 1 minute with 10 nM thrombin or 2.5 *μ*M PAR1-AP. PPACK, calcium chelators, and calpain inhibitors were included 30 minutes ahead of stimulation. *α*-Spectrin proteolysis into 150 kDa and 120 kDa fragments was assessed by Western blot. (a) Thrombin stimulates *α*-spectrin proteolysis by ~*250*% in a specific manner prevented by PPACK (25 *μ*M). 10% FBS was included as positive control. (b) Thrombin effect was mimicked by 2.5 *μ*M PAR1-AP. (c) Chelation of intracellular Ca^2+^ (10 *μ*M BAPTA-AM), calpastatin (1 *μ*M), and PD150606 (100 *μ*M) prevented calpain activation. Results are expressed as percentage of *α*-spectrin proteolysis compared to nonstimulated cells (- Control). Data are the mean ± SEM of three independent experiments. Multiple comparison ANOVA and Tukey's test: *α* = 0.001 (*∗∗∗∗*) or *α* = 0.01 (*∗∗∗*) referred to negative control. Or *α* = 0.001 (@@@@) referred to thrombin stimulation.

As shown in Figure 5(a), thrombin stimulation induced the proteolysis of *α*-spectrin 250 kDa subunit into ~150 kDa and ~120 kDa fragments. This effect appeared to be thrombin-specific, prevented by PPACK (Figure 5(a)). Thrombin-induced *α*-spectrin proteolysis was mimicked by PAR1-AP (Figure 5(b)). The specificity of *α*-spectrin degradation by calpain was confirmed by the blockage of the effect by the calpain inhibitors calpastatin and PD150606 and by the calcium chelator BAPTA-AM (Figure 5(c)). These results indicate that thrombin-induced* [Ca*^*2+*^*]i* increase activates calpain-mediated proteolysis of *α*-spectrin through the activation of PAR1.

### 3.6. Thrombin Decreases PAR1 Membrane Expression through PAR1-Induced Calpain Activation

**Figure 6:**
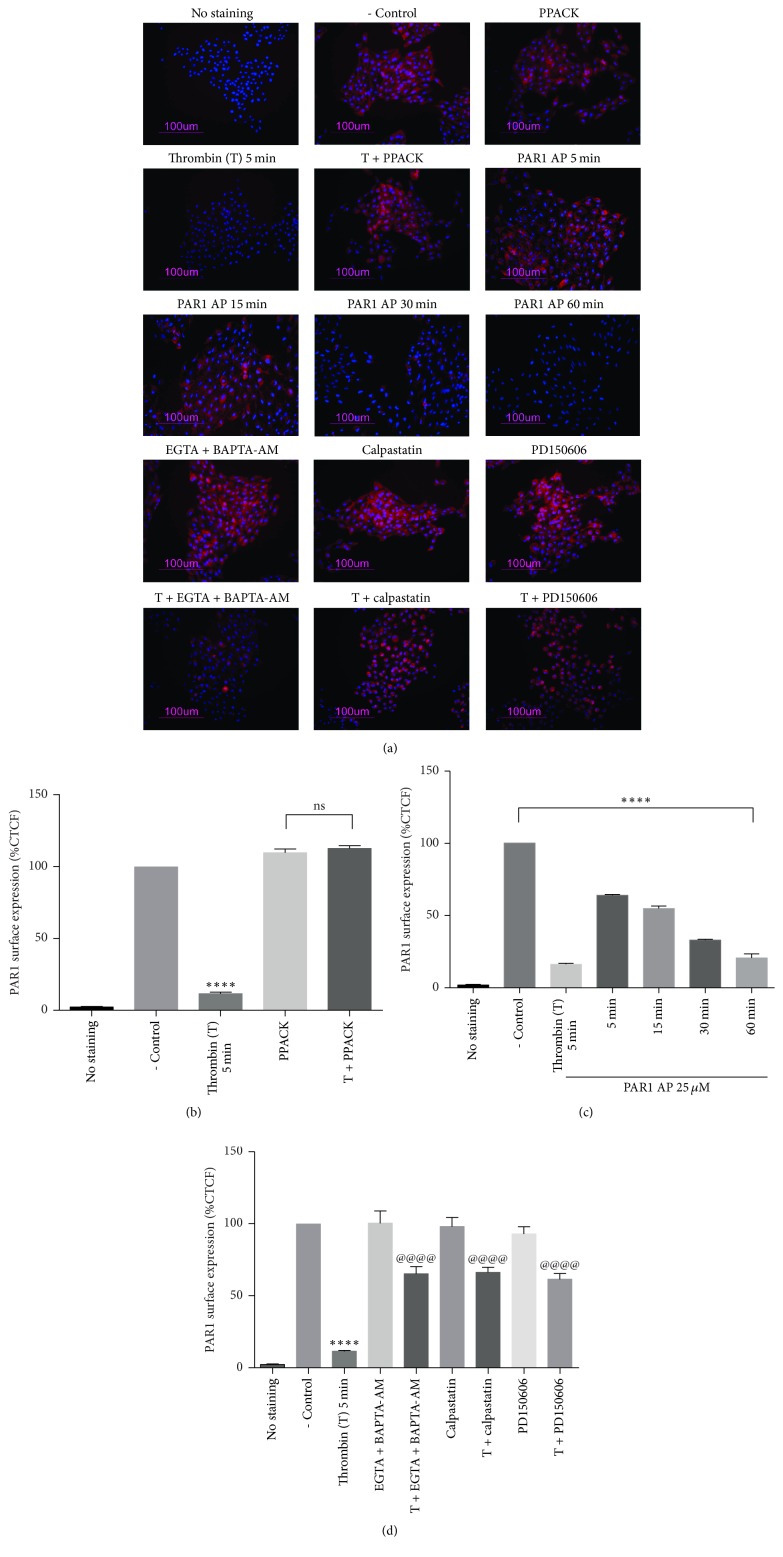
Thrombin stimulation decreases PAR1 membrane expression through calpain activation. Cells were serum-deprived for 24 hours prior to stimulation with thrombin or PAR1-AP. PAR1 membrane expression was assessed by epifluorescence microscopy. (a) PAR1 (CY3; red) and cell nuclei (Hoechst; blue) merged images. (b) Quantitative analysis of epifluorescence images shown in (a). Thrombin (10 nM) decreases PAR1 membrane expression through a specific process prevented by PPACK (25 *μ*M). (c) Stimulation with PAR1-AP (25 *μ*M) for 60 min decreases PAR1 membrane expression to the same extent as thrombin stimulation for 5 min. (d) Thrombin-induced decrease in PAR1 membrane expression requires calcium-dependent activation of calpain. Thrombin-induced decrease in PAR1 membrane expression is prevented by the joint addition of BAPTA-AM (10 *μ*M) and EGTA (0.25 mM), by calpastatin (1 *μ*M) and by PD150606 (100 *μ*M). Results are expressed as the Corrected Total Cell Fluorescence (CTCF) compared to nonstimulated cells (- Control). Data are the mean ± SEM of three independent experiments. Multiple comparison ANOVA and Tukey's test: *α* = 0.001 (*∗∗∗∗*) referred to negative control. Or *α* = 0.001 (@@@@) referred to thrombin stimulation.

**Figure 7:**
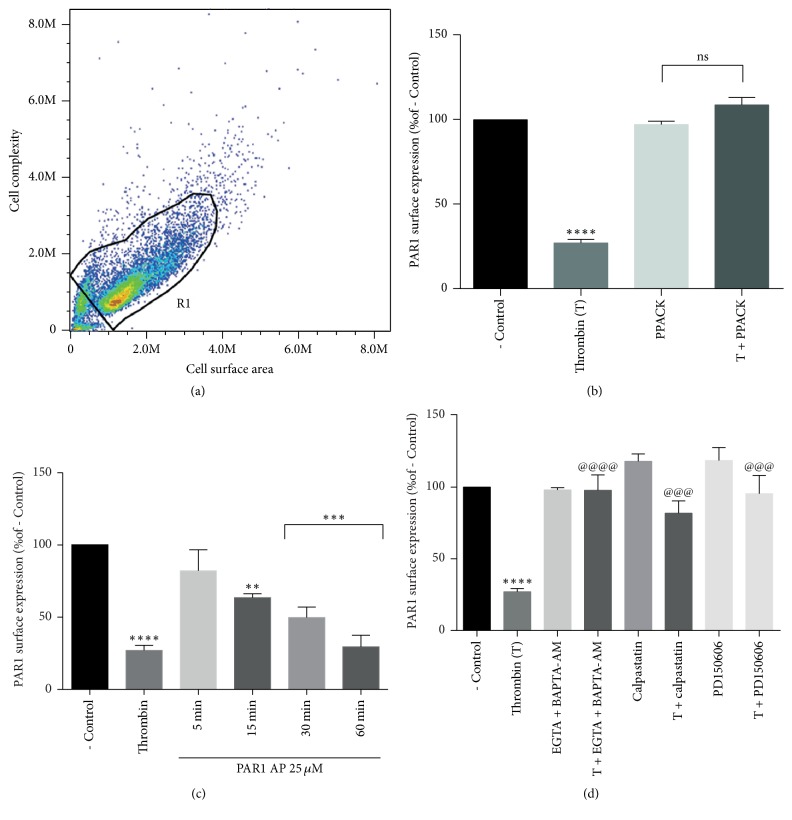
Stimulation of living cells by thrombin decreases PAR1 membrane expression through a *Ca*^2+^*- *and calpain-dependent process. Cells were serum-deprived for 24 hours prior to stimulation with thrombin or PAR1-AP. The decrease in thrombin-induced PAR1 membrane expression measured in fixed preparations (Figure 6) was confirmed in living cells by flow cytometry. (a) Cell population selected for analysis. (b) Thrombin (10 nM) stimulation decreases PAR1 membrane expression by a specific process prevented by PPACK (25 *μ*M). (c) PAR1-AP (25 *μ*M) induces time-dependent decrease in PAR1 membrane expression. (d) Thrombin induces the decrease in PAR1 membrane expression through the calcium-dependent activation of calpain. This effect was prevented by the joint addition of BAPTA-AM (10 *μ*M) and EGTA (0.25 mM), calpastatin (1 *μ*M), or PD150606 (100 *μ*M). Results are expressed as a percent of fluorescence in nonstimulated cells (- Control). Data are the mean ± SEM of three independent experiments. Multiple comparison ANOVA and Tukey's test: *α* = 0.001 (*∗∗∗∗*), *α* = 0.01 (*∗∗∗*), or *α* = 0.1 (*∗∗*) referred to negative control. And *α* = 0.001 (@@@@) or *α* = 0.01 (@@@) referred to thrombin stimulation.

The effect of thrombin-induced calpain activation on PAR1 plasma membrane expression was investigated using epifluorescence microscopy (Figure 6) and flow cytometry (Figure 7). Epifluorescence analysis in Figure 6(b) shows that stimulation with 10 nM thrombin for 5 min significantly decreased PAR1 expression at the plasma membrane; this effect was prevented by the thrombin inhibitor PPACK. To determine if thrombin effect on PAR1 membrane expression is mediated by the activation of PAR1, we analyzed the effect of PAR1-AP on PAR1 membrane expression. Time-course analysis of PAR1-AP effect showed that although stimulation by 25 *μ*M PAR1-AP for 5 min did not modify PAR1 membrane expression, sustained stimulation with the agonist for 60 min decreased PAR1 expression at the membrane to the same extent as 5 min application of thrombin (Figure 6(c)). Epifluorescence analysis showed that thrombin stimulation decreased PAR1 membrane expression by 89%. This effect was significantly prevented by calcium removal (BAPTA-AM plus EGTA) or by the inhibition of calpain by the inclusion of calpastatin or PD150606 (Figure 6(d)). In order to discard a nonspecific effect of fixation in epifluorescence analysis, PAR1 membrane expression was assessed in living cells using flow cytometry. Results from epifluorescence analysis were precisely confirmed by flow cytometry experiments. Figure 7 shows that stimulation by thrombin (Figure 7(b)) or by PAR1-AP (Figure 7(c)) decreases PAR1 membrane expression by 89%. This effect was partially prevented by inclusion of EGTA and BAPTA-AM or the inhibition of calpain by calpastatin or PD150606 (Figure 7(d)). The specificity of thrombin effect was confirmed by PPACK inhibition (Figure 7(b)). These results demonstrate that thrombin induces the Ca^2+^ and calpain-dependent decrease in PAR1 membrane expression and suggest the participation of distinct thrombin-activated processes in the regulation of PAR1 membrane expression.

## 4. Discussion

The internalization and membrane recycling of native PARs is required for cell resensitization to thrombin, whereas the internalization and lysosomal degradation of activated PARs are important for the termination of G protein signaling [11, 12]. However, the mechanisms that control PAR1 inactivation are still not clear. Thrombin has been shown to activate PAR1 in RPE cells, which promotes the epithelial-mesenchymal transformation, proliferation, and migration of RPE cells, processes involved in the development of proliferative eye diseases leading to blindness [3]. Using human-derived ARPE-19 cells as a model for RPE, in the present study we investigated the mechanism responsible for the termination of thrombin-induced PAR1 signaling and demonstrated that activation of PAR1 by thrombin decreases PAR1 membrane expression through the Ca^2+^-dependent activation of calpain. Our results suggest a novel regulatory mechanism by which activation of PAR1 controls its own availability at the plasma membrane.

We demonstrated that thrombin induces calpain activation through a specific process prevented by PPACK and identified to PAR1 as the receptor responsible for this effect (Figure 4(a)). As expected, thrombin-induced calpain activation required* [Ca*^*+2*^*]i* and was prevented by the specific calpain inhibitors calpastatin and PD150606 (Figure 4(b)). In order to establish a role for calpain in PAR1 membrane decrease, we examined thrombin effect on the cleavage of the endogenous *μ*-calpain substrate *α*-spectrin, required for clathrin-mediated endocytosis [39, 40]. Thrombin and PAR1-AP were shown to increase *α*-spectrin degradation (Figures 5(a) and 5(b)), indicating that PAR1 is responsible for thrombin-induced activation of *μ*-calpain and the subsequent degradation of *α*-spectrin (Figure 5(c)).

Thrombin-induced* [Ca*^*2+*^*]i* mobilization in RPE cells has been reported [32, 33]. Since calpain is a Ca^2+^ activated protease, we showed that thrombin induces the specific, dose-dependent increase in* [Ca*^*2+*^*]i* with an Ec_50_ of 0.55 nM (Figure 1(c)). In agreement with previous findings [8],* [Ca*^*2+*^*]i* elevation was mildly decreased in Ca^2+^-free medium (20%) and significantly inhibited by thapsigargin (80%; Figure 3), indicating that the main source of* [Ca*^*2+*^*]i* rise is Ca^2+^ release from internal stores, although Ca^2+^ influx is involved to a minor extent. Interestingly, La^3+^ inhibition of thrombin- and PAR1-induced* [Ca*^*2+*^*]i* increase to the same extent as EGTA (20%) suggests that Ca^2+^ entry through Orai-containing or voltage-gated Ca^2+^ channels could account for thrombin-induced extracellular Ca^2+^ entry (Figure 3) [41–43]. We identified PAR1 as the main receptor responsible for thrombin-induced* [Ca*^*2+*^*]i* increase, since PAR1-AP elicited* [Ca*^*2+*^*]i* increase equivalent to that induced by thrombin (Figure 2), whereas stimulation by PAR3-AP or PAR4-AP did not. These results are in agreement with findings in epithelial cells [44, 45], although in contrast with a previous report showing the increase in* [Ca*^*2+*^*]i* induced by PAR4 stimulation in ARPE-19 cells [33]. This discrepancy could be ascribed to the use by Narayan et al. (2010) of a synthetic PAR4-AP 10 times more potent than the endogenous agonist peptide used in the present work [46].

The main objective of this work was to investigate the effect of thrombin on PAR1 membrane expression and the role of calpain in the control of this process. Our results showed that the activation of PAR1 by thrombin induces a significant decrease in PAR1 membrane expression in RPE cells, via the Ca^2+^-dependent activation of *μ*-calpain and the subsequent proteolysis of *α*-spectrin by this enzyme. Since calpains are involved in the formation of coated vesicles and *α*-spectrin degradation is essential for the endocytic process, our data indicate that PAR1 signals to promote the internalization of PAR1 carried by clathrin-coated pits [47]. Nevertheless, epifluorescence and flow cytometry analysis in Figures 6(d) and 7(d) clearly showed that calcium withdrawal or calpain inhibition only partially prevents thrombin-induced reduction in PAR1 membrane expression, suggesting that calcium-dependent calpain activation is not the only mechanism involved in thrombin-induced decrease in PAR1 membrane expression. Among the* [Ca*^*2+*^*]i*- and calpain-independent mechanisms shown to regulate PAR1 membrane expression, the N-linked glycosylation of PAR-1 second extracellular loop is known to regulate the internalization of this receptor [48]. Also on this line, Bicaudal D1 (BicD1) has been identified from a screen of an embryonic cDNA library as an adapter molecule directly interacting with the C-terminal cytoplasmic domain of PAR1, involved in the transport of PAR1 from the plasma membrane to endosomal vesicles. In fact, silencing of BicD1 expression impairs endocytosis of PAR1 [49]. However, the possible effect of thrombin on the activation/regulation of these processes remains to be explored.

We here identified a novel mechanism through which thrombin activation of PAR1 autoregulates its membrane expression through the increase in* [Ca*^*2+*^*]i*, the activation of calpain by calcium, and the degradation of *α*-spectrin by calpain. Together with the present results, the future definition of additional thrombin-activated pathways involved in the regulation of PAR1 membrane expression may provide information aimed at preventing thrombin-mediated RPE cell transformation involved in proliferative retinopathies.

## References

[1] Strauss O. (2005). The retinal pigment epithelium in visual function. *Physiological Reviews*.

[2] Bastiaans J., van Meurs J. C., Mulder V. C. (2014). The Role of Thrombin in Proliferative Vitreoretinopathy. *Investigative Opthalmology & Visual Science*.

[3] Nagasaki H., Shinagawa K., Mochizuki M. (1998). Risk factors for proliferative vitreoretinopathy. *Progress in Retinal and Eye Research*.

[4] Mihara K., Ramachandran R., Saifeddine M. (2016). Thrombin-mediated direct activation of proteinase-activated Receptor-2: Another target for thrombin signaling. *Molecular Pharmacology*.

[5] Ossovskaya V. S., Bunnett N. W. (2004). Protease-activated receptors: contribution to physiology and disease. *Physiological Reviews*.

[6] Vu T.-K. H., Hung D. T., Wheaton V. I., Coughlin S. R. (1991). Molecular cloning of a functional thrombin receptor reveals a novel proteolytic mechanism of receptor activation. *Cell*.

[7] Vu T.-K. H., Wheaton V. I., Hung D. T., Charo I., Coughlin S. R. (1991). Domains specifying thrombin-receptor interaction. *Nature*.

[8] Chun J., Hla T., Spiegel S., Moolenaar W. (1994). Inhibition of thrombin receptor signaling by a *G*-protein coupled receptor kinase. Functional specificity among *G*-protein coupled receptor kinases. *Journal of Biological Chemistry*.

[9] Tiruppathi C., Yan W., Sandoval R. (2000). G protein-coupled receptor kinase-5 regulates thrombin-activated signaling in endothelial cells. *Proceedings of the National Acadamy of Sciences of the United States of America*.

[10] Lohse M. J., Benovic J. L., Codina J., Caron M. G., Lefkowitz R. J. (1990). *β*-arrestin: A protein that regulates *β*-adrenergic receptor function. *Science*.

[11] Booden M. A., Eckert L. B., Der C. J., Trejo J. (2004). Persistent Signaling by Dysregulated Thrombin Receptor Trafficking Promotes Breast Carcinoma Cell Invasion. *Molecular and Cellular Biology*.

[12] Trejo J., Hammes S. R., Coughlin S. R. (1998). Termination of signaling by protease-activated receptor-1 is linked to lysosomal sorting. *Proceedings of the National Acadamy of Sciences of the United States of America*.

[13] Paing M. M., Johnston C. A., Siderovski D. P., Trejo J. (2006). Clathrin adaptor AP2 regulates thrombin receptor constitutive internalization and endothelial cell resensitization. *Molecular and Cellular Biology*.

[14] Paing M. M., Stutts A. B., Kohout T. A., Lefkowitz R. J., Trejo J. (2002). *β*-arrestins regulate protease-activated receptor-1 desensitization but not internalization or down-regulation. *The Journal of Biological Chemistry*.

[15] Soh U. J., Dores M. R., Chen B., Trejo J. (2010). Signal transduction by protease-activated receptors. *British Journal of Pharmacology*.

[16] Chen B., Siderovski D. P., Neubig R. R., Lawson M. A., Trejo J. (2014). Regulation of protease-activated receptor 1 signaling by the adaptor protein complex 2 and R4 subfamily of regulator of G protein signaling proteins. *The Journal of Biological Chemistry*.

[17] Chen B., Dores M. R., Grimsey N., Canto I., Barker B. L., Trejo J. (2011). Adaptor Protein complex-2 (AP-2) and epsin-1 mediate protease-activated receptor-1 internalization via phosphorylation- and ubiquitination-dependent sorting signals. *The Journal of Biological Chemistry*.

[18] Goll D. E., Thompson V. F., Li H. Q., Wei W., Cong J. Y. (2003). The calpain system. *Physiological Reviews*.

[19] Ohno S., Emori Y., Imajoh S., Kawasaki H., Kisaragi M., Suzuki K. (1984). Evolutionary origin of a calcium-dependent protease by fusion of genes for a thiol protease and a calcium-binding protein?. *Nature*.

[20] Huang Y., Wang K. K. W. (2001). The calpain family and human disease. *Trends in Molecular Medicine*.

[21] Glass J. D., Culver D. G., Levey A. I., Nash N. R. (2002). Very early activation of m-calpain in peripheral nerve during Wallerian degeneration. *Journal of the Neurological Sciences*.

[22] Zimmerman U.-J. P., Boring L., Pak U. H., Mukerjee N., Wang K. K. W. (2000). The calpain small subunit gene is essential: Its inactivation results in embryonic lethality. *IUBMB Life*.

[23] Khorchid A., Ikura M. (2002). How calpain is activated by calcium. *Nature Structural & Molecular Biology*.

[24] Sato K., Saito Y., Kawashima S. (1995). Identification and Characterization of Membrane‐Bound Calpains in Clathrin‐Coated Vesicles from Bovine Brain. *European Journal of Biochemistry*.

[25] Maemoto Y., Ono Y., Kiso S. (2014). Involvement of calpain-7 in epidermal growth factor receptor degradation via the endosomal sorting pathway. *FEBS Journal*.

[26] Rudinskiy N., Grishchuk Y., Vaslin A. (2009). Calpain hydrolysis of *α*- and *β*2-adaptins decreases clathrin-dependent endocytosis and may promote neurodegeneration. *The Journal of Biological Chemistry*.

[27] Averna M., Stifanese R., Grosso R. (2010). Role of calpain in the regulation of CFTR (cystic fibrosis transmembrane conductance regulator) turnover. *Biochemical Journal*.

[28] Kamal A., Ying Y.-S., Anderson R. G. W. (1998). Annexin VI-mediated loss of spectrin during coated pit budding is coupled to delivery of LDL to lysosomes. *The Journal of Cell Biology*.

[29] Czogalla A., Sikorski A. F. (2005). Spectrin and calpain: A 'target' and a 'sniper' in the pathology of neuronal cells. *Cellular and Molecular Life Sciences*.

[30] Seyb K., Ni J., Huang M., Schuman E., Michaelis M. L., Glicksman M. A. (2007). Screen for calpain inhibitors using a cell - based , high - throughput assay calpain assay. *Cell Notes*.

[31] Hernańdez-Cruz A., Escobar A. L., Jiménez N. (1997). Ca2+-induced Ca2+ release phenomena in mammalian sympathetic neurons are critically dependent on the rate of rise of trigger Ca2+. *The Journal of General Physiology*.

[32] Catalioto R.-M., Valenti C., Maggi C. A., Giuliani S. (2015). Enhanced Ca^2+^ response and stimulation of prostaglandin release by the bradykinin B2 receptor in human retinal pigment epithelial cells primed with proinflammatory cytokines. *Biochemical Pharmacology*.

[33] Narayan S., Prasanna G., Tchedre K., Krishnamoorthy R., Yorio T. (2010). Thrombin-induced endothelin-1 synthesis and secretion in retinal pigment epithelial cells is Rho kinase dependent. *Journal of Ocular Pharmacology and Therapeutics*.

[34] O’Brien M., Scurria M., Rashka K., Daily B., Riss T. (2005). A bioluminescent assay for calpain activity. *Promega Notes*.

[35] Bennett V. (1990). Spectrin-based membrane skeleton: A multipotential adaptor between plasma membrane and cytoplasm. *Physiological Reviews*.

[36] Löfvenberg L., Backman L. (1999). Calpain-induced proteolysis of *β*-spectrins. *FEBS Letters*.

[37] Becker P. S., Schwartz M. A., Morrow J. S., Lux S. E. (1990). Radiolabel‐transfer cross‐linking demonstrates that protein 4.1 binds to the N‐terminal region of *β* spectrin and to actin in binary interactions. *European Journal of Biochemistry*.

[38] Hu R.-J., Bennett V. (1991). In vitro proteolysis of brain spectrin by calpain I inhibits association of spectrin with ankyrin-independent membrane binding site(s). *The Journal of Biological Chemistry*.

[39] Lambert M. W. (2015). Functional Significance of Nuclear *α* Spectrin. *Journal of Cellular Biochemistry*.

[40] Zhang P., Sridharan D., Lambert M. W. (2010). Knockdown of *μ*-calpain in fanconi anemia, FA-A, cells by siRNA restores *α*iI spectrin levels and corrects chromosomal instability and defective DNA interstrand cross-link repair. *Biochemistry*.

[41] Cordeiro S., Strauss O. (2011). Expression of Orai genes and ICRAC activation in the human retinal pigment epithelium. *Graefe's Archive for Clinical and Experimental Ophthalmology*.

[42] Wimmers S., Coeppicus L., Rosenthal R., Strauß O. (2008). Expression profile of voltage-dependent Ca2+ channel subunits in the human retinal pigment epithelium. *Graefe's Archive for Clinical and Experimental Ophthalmology*.

[43] Yang I.-H., Tsai Y.-T., Chiu S.-J. (2013). Involvement of STIM1 and Orai1 in EGF-mediated cell growth in retinal pigment epithelial cells. *Journal of Biomedical Science*.

[44] Ostrowska E., Reiser G. (2008). The protease-activated receptor-3 (PAR-3) can signal autonomously to induce interleukin-8 release. *Cellular and Molecular Life Sciences*.

[45] Seminario-Vidal L., Kreda S., Jones L. (2009). Thrombin promotes release of ATP from lung epithelial cells through coordinated activation of Rho- and Ca^2+^-dependent signaling pathways. *The Journal of Biological Chemistry*.

[46] Faruqi T. R., Weiss E. J., Shapiro M. J., Huang W., Coughlin S. R. (2000). Structure-function analysis of protease-activated receptor 4 thetered Ligand peptides. Determinants of specificity and utility in assays of receptor function. *The Journal of Biological Chemistry*.

[47] Sato K., Hattori S., Irie S., Sorimachi H., Inomata M., Kawashima S. (2004). Degradation of fodrin by m-calpain in fibroblasts adhering to fibrillar collagen I gel. *The Journal of Biochemistry*.

[48] Soto A. G., Trejo J. (2010). N-linked glycosylation of protease-activated receptor-1 second extracellular loop: A critical determinant for ligand-induced receptor activation and internalization. *The Journal of Biological Chemistry*.

[49] Swift S., Xu J., Trivedi V. (2010). A novel protease-activated receptor-1 interactor, bicaudal D1, regulates G protein signaling and internalization. *The Journal of Biological Chemistry*.

